# Retraction: Huang, L., Wang, L., Wang, L., Wu, Z., Jiang, F., Li, Q., & Wang, S. MicroRNA let‐7b Inhibits Proliferation and Induces Apoptosis of Castration‐Resistant Prostate Cancer Cells by Blocking the Ras/Rho Signaling Pathway via NRAS, *Clin* *. Trans. Sci.* https://ascpt.onlinelibrary.wiley.com/doi/full/10.1111/cts.12743

**DOI:** 10.1111/cts.13060

**Published:** 2022-02-13

The above article, published online on February 10, 2020 in Wiley Online Library, has been retracted by mutual agreement among the authors, the journal Editor‐in‐Chief, Dr. John Wagner, the American Society for Clinical Pharmacology and Therapeutics, and John Wiley & Sons, Inc. The retraction has been issued at the authors’ request. After questions were raised about the validity of the Western blots, the authors expanded the sample size and repeated the experiment. The results could not be confirmed and do not fully support the conclusions made in the article.

